# Prevalence of Dental Trauma in 1485 Brazilian Adolescents Aged Between 15 and 19 Years Old and Associated Factors

**DOI:** 10.3290/j.ohpd.a45100

**Published:** 2020-09-04

**Authors:** Bruno Carvalho, Híttalo Almeida, Emanuel Sávio de Souza Andrade, Patricia Zarzar, Sandra Conceição Maria Vieira, Mônica Vilela Heimer, Viviane Colares

**Affiliations:** a Master’s Student, University of Pernambuco, Brazil. Data collection and manuscript writing.; b Master’s Student, University of Pernambuco, Brazil. Article selection and manuscript review.; c Associate Professor of Oral Pathology, Dental School, University of Pernambuco, Brazil. Selection of articles and manuscript writing.; d Associate Professor of Paediatric Dentistry, Dental School, Federal University of Minas Gerais, Brazil. Manuscript review and statistical analysis.; e Associate Professor of Paediatric Dentistry, Dental School, University of Pernambuco, Brazil. Statistical analysis and revision of the manuscript.; f Associate Professor of Paediatric Dentistry, Dental School, University of Pernambuco, Brazil. Translation of the manuscript and interpretation of the data.; g Associate Professor of Paediatric Dentistry, Dental School, University of Pernambuco, Brazil. Elaboration of the methodology, statistical analysis and revision of the manuscript.

**Keywords:** dental trauma, adolescents, prevalence

## Abstract

**Purpose:**

Investigate dental trauma among adolescents aged 15–19 years and associated factors.

**Methods:**

The study was conducted in Recife, Brazil and the sample comprised 1485 adolescents of both sexes. The data was collected by clinical examination and interviews were conducted in-between classes by a single trained assessor. Two questionnaires (AUDIT and ASSIT 2.0) were used to investigate the involvement of adolescents with alcohol, tobacco and illicit drugs. The classification proposed by Andreasen was used to identify dental injuries. The sample size was calculated using 95% interval level. Pearson’s chi-square test and Fisher’s exact test were used to confirm the association between the variables.

**Results:**

The prevalence of dental injuries was 17.8%. The main causes of trauma were playing with others (20.8%) and falls (25.4%). A statistically significant percentage of adolescents reported using illicit drugs (13.9%), 15.9% used tobacco and 56.8% used alcoholic beverages. However, no statistically significant difference between the consumption of this drugs and dental injuries (p >0.005) was found.

**Conclusion:**

The prevalence of dental trauma in adolescents was high, with no association with drugs use.

Dental trauma is at present considered a public health problem not only because of its prevalence, but also because of the negative impact it has on the social life of patients.^20^

Dental traumas involve a range of factors and knowledge of these is essential for effective prevention. The dental surgeon needs to be alert to the factors related to traumas, such as the most frequent age at which they occur, how and why they occur and the most commonly affected teeth, so as to adopt adequate treatment of emergency cases and provide guidance for parents and guardians regarding the measures to be taken when a trauma occurs.^6^

In young people, the most common causes are various kinds of fall, followed by blows to the face, automobile accidents, sports injuries and assaults.^7,13^ Dental trauma is more common among male school-aged individuals, probably because they are more active and engage in rougher kinds of physical activity, such as contact sports without appropriate protection,^14^ and rough games, such as fighting, and use of toys and equipment that present more of a risk of injury. However, this difference has been decreasing, owing to greater participation of women in activities which, until recently, had been considered the preserve of males.^2,9^

Predisposing anatomical factors may also increase the likelihood of dental trauma. These include pronounced overjet and inadequate lip coverage. Overjet of more than 5 mm and the lack of lip protection are considered to be factors that significantly predispose individuals to dental trauma.^3,18^

A previously fractured tooth may lead to physical incapacity, difficulty in chewing and speaking (or both) and may cause social and psychological embarrassment, such as avoiding smiling, thereby affecting social relations.^8^

In the associated literature, there is a shortage of population-based studies investigating the prevalence of dental trauma and its aetiological factors in adolescents aged 15–19 years.^20^

The aim of this cross-sectional study is therefore to determine the prevalence of dental trauma, involvement with alcohol, tobacco, illicit drugs and related factors among individuals 15–19 years of age who attend school in the city of Recife in the Brazilian state of Pernambuco.

## Materials and Methods

This study was conducted in the city of Recife, state of Pernambuco, Brazil. The calculation of the sample size was based on the prevalence of 10.5% reported by Soriano et al (2007)^18^ in a study carried out in Recife-PE, with a sample comprising 12-year-old school children giving an initial n value of 819 individuals. As this was a conglomerate sampling model, the sample size was multiplied by 1.5 giving a total of 1475 individuals.

The city of Recife is divided into six administrative regions (RPAs) and, to ensure that the study covered the whole city, schools were selected randomly, with at least three schools for each region. Data was collected in 22 schools across the six administrative regions of the city. Fourteen of them were public schools and eight were private. The number of adolescents covered by the study was distributed according to data obtained from the Pernambuco State Education Department.

The clinical examination and interview were carried out in a classroom, under natural lighting, in a conventional chair, using personal protective equipment (PPEs) and disposable materials.

The calibration process was conducted considering the classification of dental trauma in accordance with Andreassen & Andreassen (2001).^1^ The Kappa test was conducted and interpreted using the Bulman & Osborn scale (1989). There was found to be an agreement of k = 0.95 with the gold standard and an agreement between assessors of k = 1, established by an examination being carried out with the same patient twice by the same assessor. This is considered to be a high level of reliability.

Dental traumas involving the root, the cement or both were not included in this study, as it was not possible to use X-rays. The clinical and epidemiological data were recorded on a form containing information on gender, age, type of school, schooling of parents and the presence of trauma. An interview was conducted with those individuals who showed clinical signs of trauma with detailed questions on the cause, the teeth affected, the number of teeth involved.

Lip coverage was assessed visually on the assessor’s first contact with the participant. Lip coverage was considered to be adequate when the upper lip completely covered the upper incisors in a resting position.

The measurement of the overjet was obtained using a wooden spatula and it was considered raised when it presented values higher than 3 mm. First, the participants were asked to clench their teeth. The assessor placed the wooden spatula perpendicular to the vestibular edge of the mandibular incisors and marked the limit of contact with the maxillary incisors with a pen. Then the distance was measured using a ruler and the horizontal overjet measurement was noted. The overjet was considered to be pronounced when it exceeded 3 mm.

In order to evaluate the involvement of adolescents with alcohol, tobacco and illicit drugs two questionnaires were applied: ASSIST version 2.0 to evaluate the risk of drug abuse and AUDIT to assess the risk of alcohol consumption. The questionnaires did not contain identification.

The SPSS (Statistical Package for the Social Sciences) Version 15 was used for data entry and statistical calculations. Data analysis involved absolute distributions, percentages and statistical measures: Pearson’s chi-square test was carried out and Fisher’s exact test was chosen (statistical inference techniques).

The research project was approved by the University of Pernambuco’s Committee for Ethics in Research involving Human Beings (protocol 211/09). Terms of free and informed consent were obtained from parents and guardians of the adolescents aged under 18 and from the adolescents aged 18 and 19 themselves.

## Results

The prevalence of dental trauma was 17.8%, and considering gender, the prevalence was higher among males (20.1% vs 15.9%), and this difference was statistically significant (p = 0.037). The prevalence of dental trauma varied according to age. The lowest rate was for those aged 15 (16.2%) and the highest for those aged 18 (24.1%). However, there was no statistically significant difference (p = 0.409).

A percentage of adolescents do not presented pronounced overjet and inadequate lip coverage.

Table 1 shows the distribution of adolescents analysed according to pronounced overjet, lip protection, affected teeth, number of injured teeth, how the trauma occurred, and type of trauma. Among adolescents who had suffered some type of trauma the most commonly affected teeth were 11 and 21, most of the adolescents had only one tooth affected by trauma and the most common cause was falls.

**Table 1:** Distribution of adolescents analysed according to pronounced overjet, lip protection, affected teeth, number of injured teeth, how the trauma occurred, and type of trauma

Variable	n	%
Pronounced overjet		
Yes	541	36.4
No	944	63.6
Lip protection		
Inadequate	469	31.6
Adequate	1016	68.4
TOTAL	1485	100.0
Affected teeth by trauma		
11	142	53.8
12	7	2.7
21	102	38.6
22	10	3.8
31	2	0.8
32	1	0.8
Number of injured teeth		
One	213	80.7
Two	49	18.6
Four	24	0.8
How the trauma occurred		
Playing team sports	15	5.7
Practicing martial arts	5	1.9
Playing with others	55	20.8
Traffic accidents	3	1.1
Falls	67	25.4
Being struck	8	3.0
Using teeth for something other than eating	14	5.3
Eating	11	4.2
Involvement in an act of violence	7	0.5
Do not remember	79	29.9
Type of trauma (1)		
Fracture and/or chipping of enamel	208	78.8
Fracture of enamel + dentine without exposure of pulp	76	28.8
Fracture of enamel + dentine with exposure of pulp	15	5.7
avulsion	4	1.5
Lateral luxation	3	1.1
TOTAL	264	100.0

(1): As a single patient may have had more than one type of trauma, the base for the calculation of percentages is not the total.

Table 2 shows the consumption of alcohol, tobacco, and illicit drugs, which proved to be high. However, most adolescents were classified as low risk or in abstinence.

**Table 2:** Distribution of adolescents analysed according to consumption of alcohol, tobacco and illicit drugs assessed by the ASSIST 2.0 and AUDIT questionnaires

Variable	n	%
Use of illicit drugs		
Yes	206	13.9
No	1279	86.1
Use of tobacco		
Yes	236	15.9
No	1249	84.1
Use of alcohol		
Yes	843	56.8
No	642	43.2
Risk of alcohol use (AUDIT)		
Low risk/abstinence	1279	86.1
Risk	155	10.4
Harmful use	30	2.0
Possible dependence	21	1.4
Risk of alcohol use (ASSIT 2.0)		
Indicative use of rational	1201	80.9
Indicative of abuse	283	19.0
Suggestive dependence	2	0.1
BASE	1485	100.0

Table 3 shows that there was no statistically significant association between dental trauma and lip coverage (p = 0.090); dental trauma and overjet (p = 0.213); dental trauma and consumption of illicit and licit drugs (p = 0.507), (p = 0.390), respectively.

**Table 3:** Evaluation of the occurrence of trauma according to the following characteristics: overjet, lip coverage, use of alcohol and tobacco, risk of alcohol use and illicit drug use

Variable	Prevalence of dental trauma						p value	OR (IC 95%)
	Yes		No		Total			
	n	%	n	%	N	%		
Overjet								
Yes	105	19.4	436	80.6	541	100.0	p^(1)^ = 0.213	1.19 (0.91–1.56)
No	159	16.8	785	83.2	944	100.0		1.00
Lip covered								
Inadequate	169	16.6	847	83.4	1016	100.0	p^(1)^ = 0.090	1.00
Adequate	95	20.3	374	79.7	469	100.0		1.27 (0.96–1.68)
Use of alcohol								
Yes	153	18.1	690	81.9	843	100.0	p^(1)^ = 0.668	1.06 (0.81–1.39)
No	111	17.3	531	82.7	642	100.0		1.00
Use of tobacco								
Yes	44	18.6	192	81.4	236	100.0	p^(1)^ = 0.704	1.07 (0.75–1.53)
No	220	17.6	1029	82.4	1249	100.0		1.00
Risk of alcohol use (AUDIT)								
Low risk/abstinence	223	17.4	1056	82.6	1279	100.0	p^(1)^ = 0.390	1.00
Risk/harmful/dependence	41	19.9	165	80.1	206	100.0		1.18 (0.81–1.71)
Use of illicit drug								
Yes	40	19.4	166	80.6	206	100.0	p^(1)^ = 0.507	1.14 (0.78–1.65)
No	224	17.5	1055	82.5	1279	100.0		1.00
Total	264	17.8	1221	82.2	1485	100.0		

(1): Chi-square test.

The prevalence of trauma was investigated in all regions (RPAs) of the city and there was a statistically significant difference (p = 0.007) (Table 4).

**Table 4:** Evaluation of the occurrence of trauma according to age, gender, type of school home district and mother’s education

Variable	Prevalence of trauma				TOTAL		Value of p	OR (IC 95%)
	Yes		No					
	N	%	n	%	n	%		
Age								
15	84	16.2	434	83.8	518	100.0	p^(1)^ = 0.409	1.00
16	82	17.7	380	82.3	462	100.0		1.12 (0.80–1.56)
17	61	17.8	281	82.2	342	100.0		1.12 (0.78–1.61)
18	26	24.1	82	75.9	108	100.0		1.64 (0.99–2.70)
19	11	20.0	44	80.0	55	100.0		1.29 (0.64–2.60)
Gender								
Male	133	20.1	529	79.9	662	100.0	p^(1)^ = 0.037^*^	1.33 (1.02–1.73)
Female	131	15.9	692	84.1	823	100.0		1.00
School								
Public	136	16.9	670	83.1	806	100.0	p^(1)^ = 0.321	1.00
Private	128	18.9	551	81.1	679	100.0		1.14 (0.88–1.49)
Home district								
1	29	15.6	157	84.4	186	100.0	p^(1)^ = 0.007^*^	1.00
2	52	26.5	144	73.5	196	100.0		1.95 (1.18–3.25)
3	59	15.3	327	84.7	386	100.0		0.98 (0.60–1.58)
4	49	17.3	235	82.7	284	100.0		1.13 (0.68–1.86)
5	14	11.9	104	88.1	118	100.0		0.73 (0.37–1.44)
6	61	19.4	254	80.6	315	100.0		1.30 (0.80–2.11)
Total group	264	17.8	1221	82.2	1485	100.0		
Mother’s education								
8 years	6	14.3	36	85.7	42	100.0	p^(1)^ = 0.500	1.00
>8 years	223	18.4	991	81.6	1214	100.0		1.35 (0.56–3.24)
Total group^(2)^	229	18.2	1027	81.8	1256	100.0		

(*): Unable to determine due to the occurrence of very low frequency; (1): Chi-square test; (2): Did not consider the 229 respondents who did not know the mother’s education.

## Discussion

The prevalence of dental injuries found in adolescents between 15 and 19 years was 17.8% and varies greatly from 3.16% to 22.3%. A number of factors, such as location of the research, methodology used, age group, and criteria used to classify dental trauma, may explain the difference in prevalence. The findings of this study are in accordance with those of most authors who have studied adolescents in the same age group.^2,10–12^

As in other studies^10,21^ most patients who had suffered a trauma (25.4%) reported having had a fall and 20.8% reported playing with other adolescents.

Corroborating the findings of most authors according to whom trauma is more prevalent in males,^12,13,19,21^ the present study found a prevalence of 20.1% in males compared with 15.9% in females. It has been suggested that the cause for this is that males are more likely to be involved in violent incidents than females.^5,12,19,21^

Different from other studies,^4,19^ the prevalence of dental trauma among adolescents enrolled in public schools (16.9%) and those enrolled in private schools (18.9%) was not statistically different (p = 0.321).

The socioeconomic indicators used in this research were the regions (RPAs) and school type (public or private). School type did not show statistically significant association with dental trauma (p = 0321). However, when associated with dental trauma to the different regions of the city, there was a statistically significant relationship (p = 0.007). This can be explained by socioeconomic inequality between the regions surveyed. It is noteworthy that few authors have investigated the relationship between dental trauma and socioeconomic status and the results are often conflicting. There is a need to investigate this correlation in future studies.

 An association between the prevalence of dental trauma and type of school was found, being higher in the private ones.^20^ Children coming from private schools showed a three times higher likelihood of suffering injury when compared to public school children who have the worst socioeconomic conditions. Some authors observed an association between higher levels of parental education and prevalence of dental trauma.^3,5,16,20^ Higher levels of education provide better jobs with better wages and, consequently, children of higher socioeconomic status have greater access to sports and leisure activities such as swimming pools, bicycles, skateboards, than children from disadvantaged socioeconomic conditions.

There was no association between consumption of licit and illicit drugs and dental trauma. This can be explained by a bias of information, although there was no identification on the questionnaires.

Corroborating with some authors who found no statistically significant association between the lip coverage and dental trauma,^15,16^ this study showed no association (p = 0.090). However, some authors^3,8^ have found a correlation.

There is a consensus in the literature regarding the most commonly affected tooth.^2,4,10^ In the present study, 92.4% of the dental traumas occurred in the maxillary central incisors, with the maxillary central right incisor being the one most frequently injured.

As for the type of trauma suffered by adolescents, some authors corroborate the finding that the most common kinds of injuries in the permanent incisors are fractures of the enamel and dentine, although there is some divergence in the classification systems used by the various studies in the literature.^2,4,10^

The literature presents several studies^17,22^ regarding the use of alcohol, tobacco and illicit drugs. Research on the use of psychoactive substances among school youths may cause possible information bias associated with lack of seriousness, in a hurry to finish answering, self-criticism, suspicion that school authorities may wish to have access to completed questionnaires, lack of attention or understanding. In this study the consumption of alcohol, tobacco and illicit drugs shows that: although many teenagers having used no association was found. However, when associated with dental trauma, consumption of alcohol (p = 0.668), tobacco (p = 0.704) and illicit drugs (p = 0.507), no statistically significant association in the sample was found.

## Conclusions

Based on the findings of the study, it can be concluded that the prevalence of dental trauma in adolescents was high, with no association with drug use.
